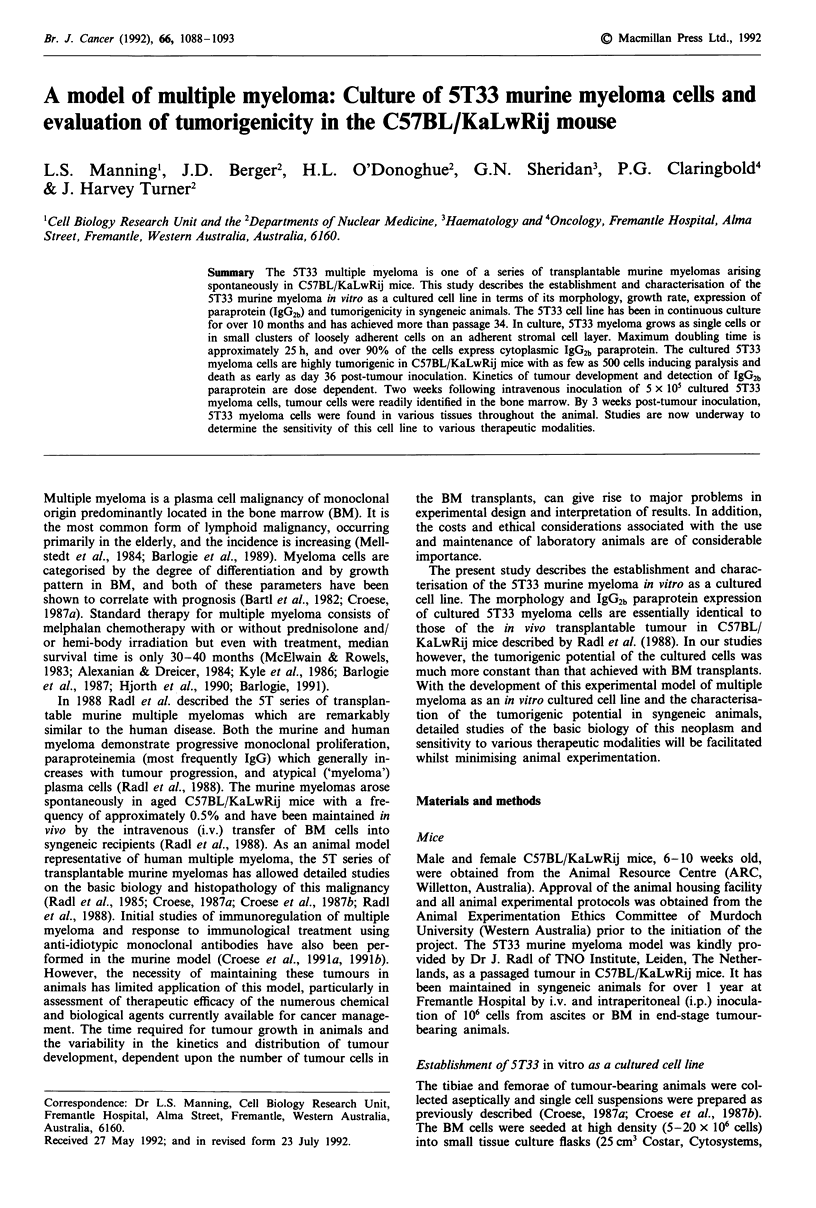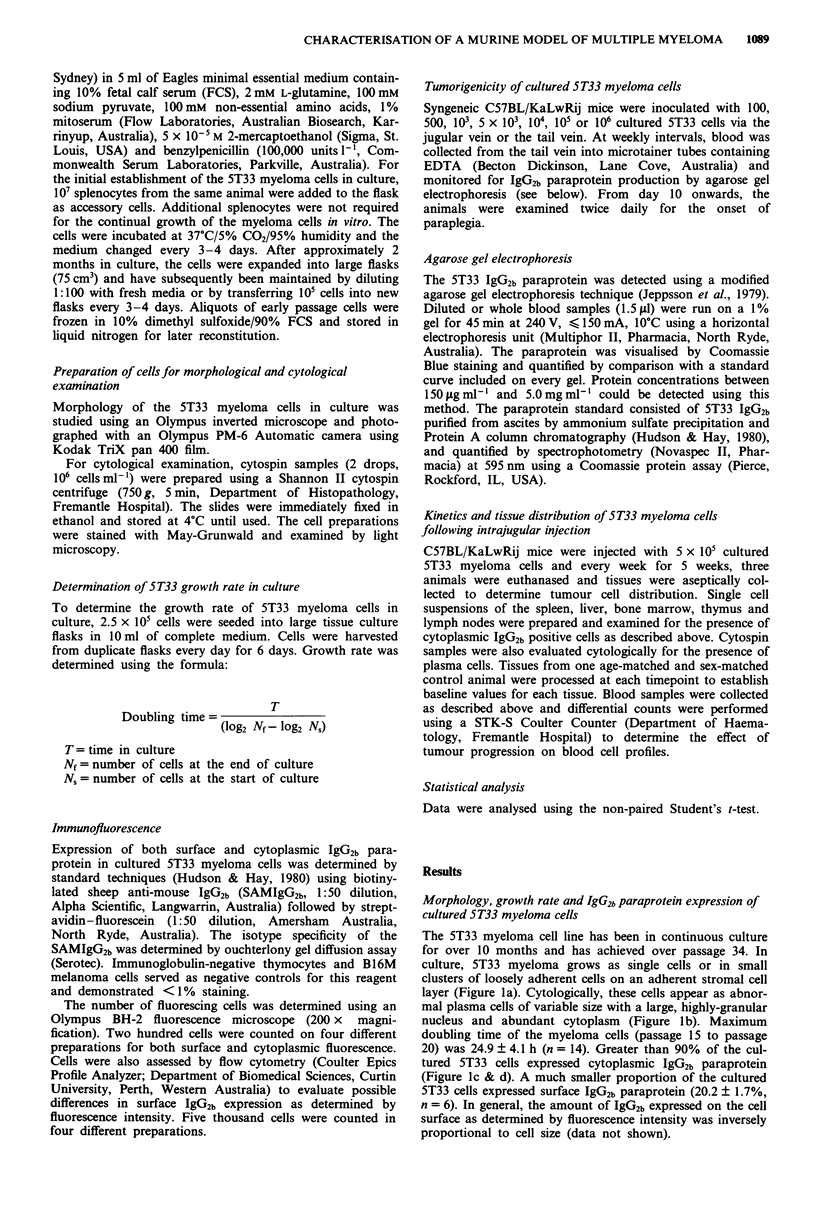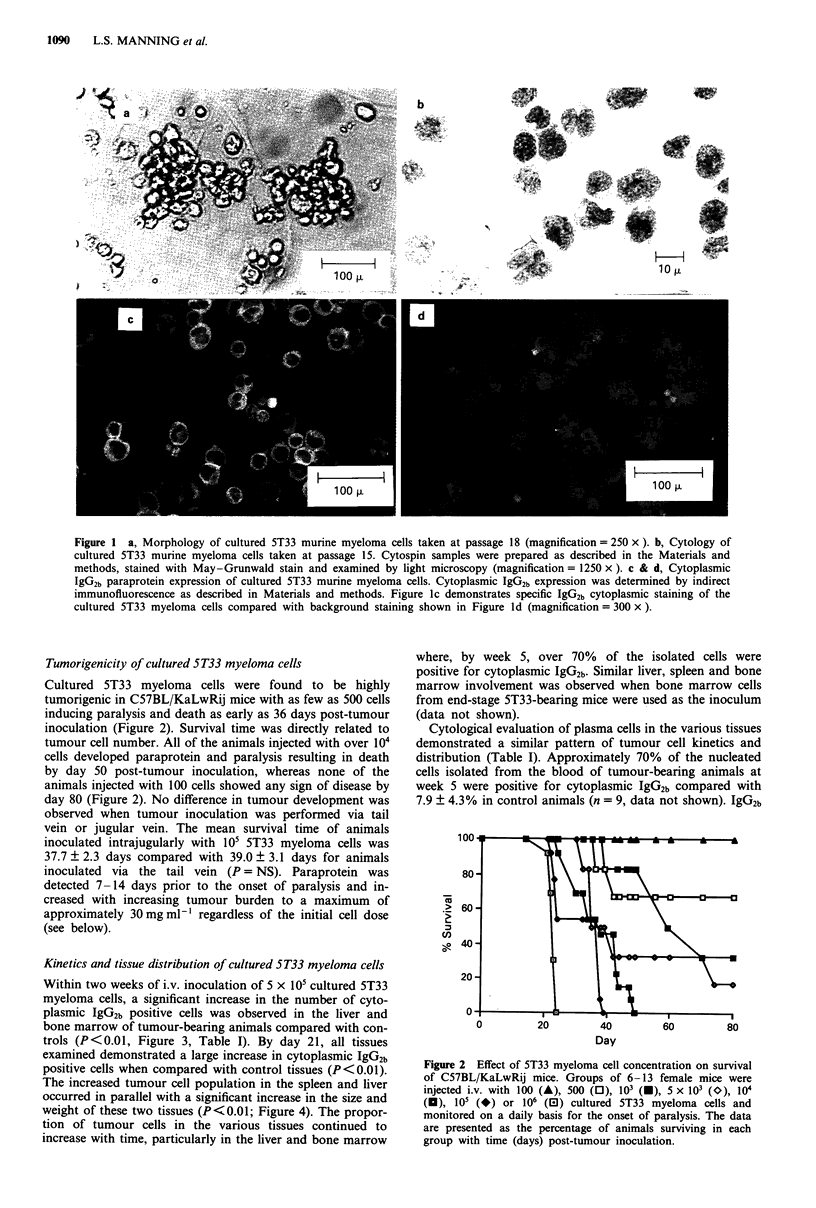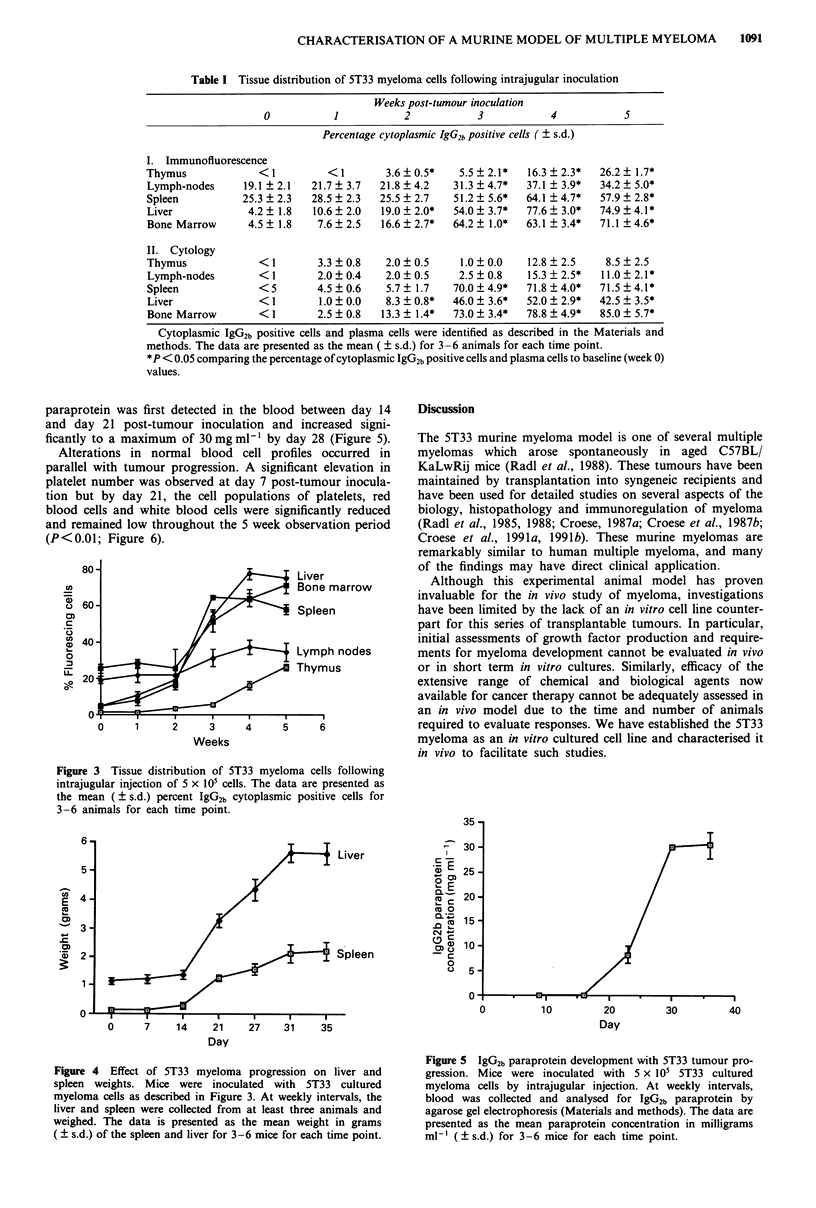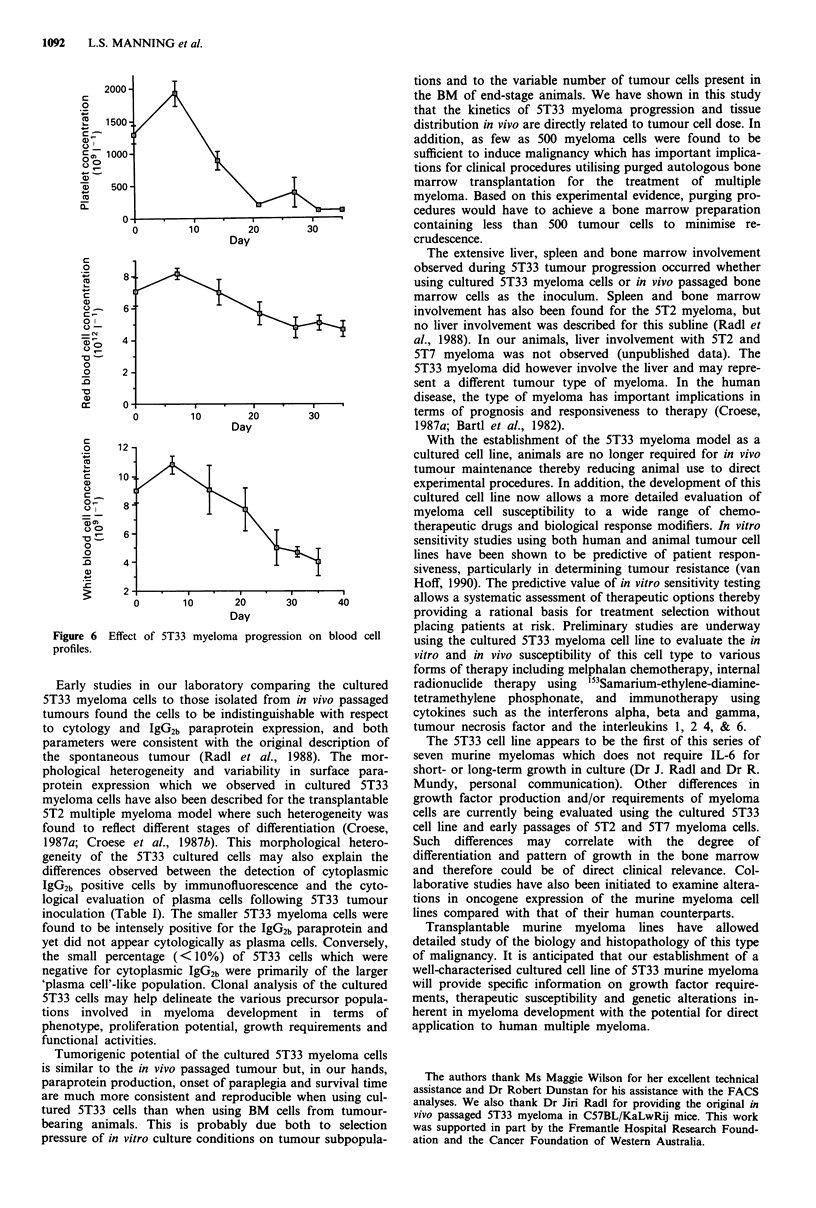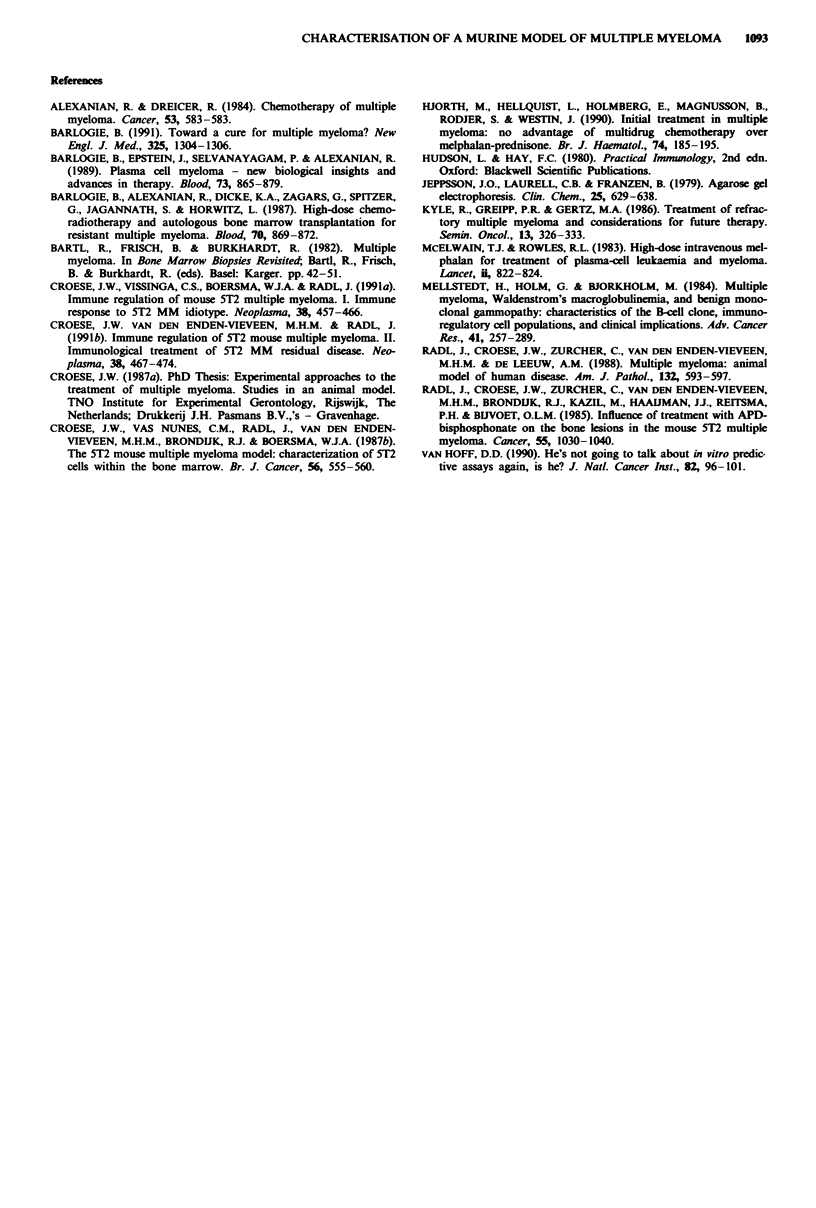# A model of multiple myeloma: culture of 5T33 murine myeloma cells and evaluation of tumorigenicity in the C57BL/KaLwRij mouse.

**DOI:** 10.1038/bjc.1992.415

**Published:** 1992-12

**Authors:** L. S. Manning, J. D. Berger, H. L. O'Donoghue, G. N. Sheridan, P. G. Claringbold, J. H. Turner

**Affiliations:** Cell Biology Research Unit, Fremantle Hospital, Fremantle, Western Australia.

## Abstract

**Images:**


Br. J. Cancer (1992), 66, 1088-1093 © Macmillan Press Ltd., 1992

### A model of multiple myeloma: Culture of 5T33 murine myeloma cells and evaluation of tumorigenicity in the C57BL/KaLwRij mouse

L.S. Manning[1], J.D. Berger[2], H.L. O'Donoghue[2], G.N. Sheridan[3], P.G. Claringbold[4] & J. Harvey Turner[2]

[1]*Cell Biology Research Unit and the* [2]*Departments of Nuclear Medicine,* [3]*Haematology and* [4]*Oncology, Fremantle Hospital, Alma Street, Fremantle, Western Australia, Australia, 6160.*

**Summary** The 5T33 multiple myeloma is one of a series of transplantable murine myelomas arising spontaneously in C57BL/KaLwRij mice. This study describes the establishment and characterisation of the 5T33 murine myeloma *in vitro* as a cultured cell line in terms of its morphology, growth rate, expression of paraprotein ($IgG_{2b}$) and tumorigenicity in syngeneic animals. The 5T33 cell line has been in continuous culture for over 10 months and has achieved more than passage 34. In culture, 5T33 myeloma grows as single cells or in small clusters of loosely adherent cells on an adherent stromal cell layer. Maximum doubling time is approximately 25 h, and over 90% of the cells express cytoplasmic $IgG_{2b}$ paraprotein. The cultured 5T33 myeloma cells are highly tumorigenic in C57BL/KaLwRij mice with as few as 500 cells inducing paralysis and death as early as day 36 post-tumour inoculation. Kinetics of tumour development and detection of $IgG_{2b}$ paraprotein are dose dependent. Two weeks following intravenous inoculation of $5 \times 10^5$ cultured 5T33 myeloma cells, tumour cells were readily identified in the bone marrow. By 3 weeks post-tumour inoculation, 5T33 myeloma cells were found in various tissues throughout the animal. Studies are now underway to determine the sensitivity of this cell line to various therapeutic modalities.

Multiple myeloma is a plasma cell malignancy of monoclonal origin predominantly located in the bone marrow (BM). It is the most common form of lymphoid malignancy, occurring primarily in the elderly, and the incidence is increasing (Mellstedt *et al.*, 1984; Barlogie *et al.*, 1989). Myeloma cells are categorised by the degree of differentiation and by growth pattern in BM, and both of these parameters have been shown to correlate with prognosis (Bartl *et al.*, 1982; Croese, 1987*a*). Standard therapy for multiple myeloma consists of melphalan chemotherapy with or without prednisolone and/or hemi-body irradiation but even with treatment, median survival time is only 30–40 months (McElwain & Rowels, 1983; Alexanian & Dreicer, 1984; Kyle *et al.*, 1986; Barlogie *et al.*, 1987; Hjorth *et al.*, 1990; Barlogie, 1991).

In 1988 Radl *et al.* described the 5T series of transplantable murine multiple myelomas which are remarkably similar to the human disease. Both the murine and human myeloma demonstrate progressive monoclonal proliferation, paraproteinemia (most frequently IgG) which generally increases with tumour progression, and atypical ('myeloma') plasma cells (Radl *et al.*, 1988). The murine myelomas arose spontaneously in aged C57BL/KaLwRij mice with a frequency of approximately 0.5% and have been maintained *in vivo* by the intravenous (i.v.) transfer of BM cells into syngeneic recipients (Radl *et al.*, 1988). As an animal model representative of human multiple myeloma, the 5T series of transplantable murine myelomas has allowed detailed studies on the basic biology and histopathology of this malignancy (Radl *et al.*, 1985; Croese, 1987*a*; Croese *et al.*, 1987*b*; Radl *et al.*, 1988). Initial studies of immunoregulation of multiple myeloma and response to immunological treatment using anti-idiotypic monoclonal antibodies have also been performed in the murine model (Croese *et al.*, 1991*a*, 1991*b*). However, the necessity of maintaining these tumours in animals has limited application of this model, particularly in assessment of therapeutic efficacy of the numerous chemical and biological agents currently available for cancer management. The time required for tumour growth in animals and the variability in the kinetics and distribution of tumour development, dependent upon the number of tumour cells in the BM transplants, can give rise to major problems in experimental design and interpretation of results. In addition, the costs and ethical considerations associated with the use and maintenance of laboratory animals are of considerable importance.

The present study describes the establishment and characterisation of the 5T33 murine myeloma *in vitro* as a cultured cell line. The morphology and $IgG_{2b}$ paraprotein expression of cultured 5T33 myeloma cells are essentially identical to those of the *in vivo* transplantable tumour in C57BL/KaLwRij mice described by Radl *et al.* (1988). In our studies however, the tumorigenic potential of the cultured cells was much more constant than that achieved with BM transplants. With the development of this experimental model of multiple myeloma as an *in vitro* cultured cell line and the characterisation of the tumorigenic potential in syngeneic animals, detailed studies of the basic biology of this neoplasm and sensitivity to various therapeutic modalities will be facilitated whilst minimising animal experimentation.

### Materials and methods

### *Mice*

Male and female C57BL/KaLwRij mice, 6–10 weeks old, were obtained from the Animal Resource Centre (ARC, Willetton, Australia). Approval of the animal housing facility and all animal experimental protocols was obtained from the Animal Experimentation Ethics Committee of Murdoch University (Western Australia) prior to the initiation of the project. The 5T33 murine myeloma model was kindly provided by Dr J. Radl of TNO Institute, Leiden, The Netherlands, as a passaged tumour in C57BL/KaLwRij mice. It has been maintained in syngeneic animals for over 1 year at Fremantle Hospital by i.v. and intraperitoneal (i.p.) inoculation of $10^6$ cells from ascites or BM in end-stage tumour-bearing animals.

### *Establishment of 5T33* in vitro *as a cultured cell line*

The tibiae and femorae of tumour-bearing animals were collected aseptically and single cell suspensions were prepared as previously described (Croese, 1987*a*; Croese *et al.*, 1987*b*). The BM cells were seeded at high density ($5-20 \times 10^6$ cells) into small tissue culture flasks ($25 cm^3$ Costar, Cytosystems,

Correspondence: Dr L.S. Manning, Cell Biology Research Unit, Fremantle Hospital, Alma Street, Fremantle, Western Australia, Australia, 6160.
Received 27 May 1992; and in revised form 23 July 1992.

Sydney) in 5 ml of Eagles minimal essential medium containing 10% fetal calf serum (FCS), 2 mM L-glutamine, 100 mM sodium pyruvate, 100 mM non-essential amino acids, 1% mitoserum (Flow Laboratories, Australian Biosearch, Karrinyup, Australia), $5 \times 10^{-5}$ M 2-mercaptoethanol (Sigma, St. Louis, USA) and benzylpenicillin (100,000 units $l^{-1}$, Commonwealth Serum Laboratories, Parkville, Australia). For the initial establishment of the 5T33 myeloma cells in culture, $10^7$ splenocytes from the same animal were added to the flask as accessory cells. Additional splenocytes were not required for the continual growth of the myeloma cells *in vitro*. The cells were incubated at 37°C/5% $CO_2$/95% humidity and the medium changed every 3–4 days. After approximately 2 months in culture, the cells were expanded into large flasks (75 $cm^3$) and have subsequently been maintained by diluting 1:100 with fresh media or by transferring $10^5$ cells into new flasks every 3–4 days. Aliquots of early passage cells were frozen in 10% dimethyl sulfoxide/90% FCS and stored in liquid nitrogen for later reconstitution.

### *Preparation of cells for morphological and cytological examination*

Morphology of the 5T33 myeloma cells in culture was studied using an Olympus inverted microscope and photographed with an Olympus PM-6 Automatic camera using Kodak TriX pan 400 film.

For cytological examination, cytospin samples (2 drops, $10^6$ cells $ml^{-1}$) were prepared using a Shannon II cytospin centrifuge (750 *g*, 5 min, Department of Histopathology, Fremantle Hospital). The slides were immediately fixed in ethanol and stored at 4°C until used. The cell preparations were stained with May-Grunwald and examined by light microscopy.

### *Determination of 5T33 growth rate in culture*

To determine the growth rate of 5T33 myeloma cells in culture, $2.5 \times 10^5$ cells were seeded into large tissue culture flasks in 10 ml of complete medium. Cells were harvested from duplicate flasks every day for 6 days. Growth rate was determined using the formula:

$$\text{Doubling time} = \frac{T}{(\log_2 N_f - \log_2 N_s)}$$

$T$ = time in culture
$N_f$ = number of cells at the end of culture
$N_s$ = number of cells at the start of culture

### *Immunofluorescence*

Expression of both surface and cytoplasmic $IgG_{2b}$ paraprotein in cultured 5T33 myeloma cells was determined by standard techniques (Hudson & Hay, 1980) using biotinylated sheep anti-mouse $IgG_{2b}$ ($SAMIgG_{2b}$, 1:50 dilution, Alpha Scientific, Langwarrin, Australia) followed by streptavidin–fluorescein (1:50 dilution, Amersham Australia, North Ryde, Australia). The isotype specificity of the $SAMIgG_{2b}$ was determined by ouchterlony gel diffusion assay (Serotec). Immunoglobulin-negative thymocytes and B16M melanoma cells served as negative controls for this reagent and demonstrated $<1\%$ staining.

The number of fluorescing cells was determined using an Olympus BH-2 fluorescence microscope (200 × magnification). Two hundred cells were counted on four different preparations for both surface and cytoplasmic fluorescence. Cells were also assessed by flow cytometry (Coulter Epics Profile Analyzer; Department of Biomedical Sciences, Curtin University, Perth, Western Australia) to evaluate possible differences in surface $IgG_{2b}$ expression as determined by fluorescence intensity. Five thousand cells were counted in four different preparations.

### *Tumorigenicity of cultured 5T33 myeloma cells*

Syngeneic C57BL/KaLwRij mice were inoculated with 100, 500, $10^3$, $5 \times 10^3$, $10^4$, $10^5$ or $10^6$ cultured 5T33 cells via the jugular vein or the tail vein. At weekly intervals, blood was collected from the tail vein into microtainer tubes containing EDTA (Becton Dickinson, Lane Cove, Australia) and monitored for $IgG_{2b}$ paraprotein production by agarose gel electrophoresis (see below). From day 10 onwards, the animals were examined twice daily for the onset of paraplegia.

### *Agarose gel electrophoresis*

The 5T33 $IgG_{2b}$ paraprotein was detected using a modified agarose gel electrophoresis technique (Jeppsson *et al.*, 1979). Diluted or whole blood samples (1.5 µl) were run on a 1% gel for 45 min at 240 V, $\leqslant 150$ mA, 10°C using a horizontal electrophoresis unit (Multiphor II, Pharmacia, North Ryde, Australia). The paraprotein was visualised by Coomassie Blue staining and quantified by comparison with a standard curve included on every gel. Protein concentrations between 150 µg $ml^{-1}$ and 5.0 mg $ml^{-1}$ could be detected using this method. The paraprotein standard consisted of 5T33 $IgG_{2b}$ purified from ascites by ammonium sulfate precipitation and Protein A column chromatography (Hudson & Hay, 1980), and quantified by spectrophotometry (Novaspec II, Pharmacia) at 595 nm using a Coomassie protein assay (Pierce, Rockford, IL, USA).

### *Kinetics and tissue distribution of 5T33 myeloma cells following intrajugular injection*

C57BL/KaLwRij mice were injected with $5 \times 10^5$ cultured 5T33 myeloma cells and every week for 5 weeks, three animals were euthanased and tissues were aseptically collected to determine tumour cell distribution. Single cell suspensions of the spleen, liver, bone marrow, thymus and lymph nodes were prepared and examined for the presence of cytoplasmic $IgG_{2b}$ positive cells as described above. Cytospin samples were also evaluated cytologically for the presence of plasma cells. Tissues from one age-matched and sex-matched control animal were processed at each timepoint to establish baseline values for each tissue. Blood samples were collected as described above and differential counts were performed using a STK-S Coulter Counter (Department of Haematology, Fremantle Hospital) to determine the effect of tumour progression on blood cell profiles.

### *Statistical analysis*

Data were analysed using the non-paired Student's *t*-test.

### Results

### *Morphology, growth rate and $IgG_{2b}$ paraprotein expression of cultured 5T33 myeloma cells*

The 5T33 myeloma cell line has been in continuous culture for over 10 months and has achieved over passage 34. In culture, 5T33 myeloma grows as single cells or in small clusters of loosely adherent cells on an adherent stromal cell layer (Figure 1a). Cytologically, these cells appear as abnormal plasma cells of variable size with a large, highly-granular nucleus and abundant cytoplasm (Figure 1b). Maximum doubling time of the myeloma cells (passage 15 to passage 20) was $24.9 \pm 4.1$ h ($n = 14$). Greater than 90% of the cultured 5T33 cells expressed cytoplasmic $IgG_{2b}$ paraprotein (Figure 1c & d). A much smaller proportion of the cultured 5T33 cells expressed surface $IgG_{2b}$ paraprotein ($20.2 \pm 1.7\%$, $n = 6$). In general, the amount of $IgG_{2b}$ expressed on the cell surface as determined by fluorescence intensity was inversely proportional to cell size (data not shown).

**Figure 1** a, Morphology of cultured 5T33 murine myeloma cells taken at passage 18 (magnification = 250 ×). b, Cytology of cultured 5T33 murine myeloma cells taken at passage 15. Cytospin samples were prepared as described in the Materials and methods, stained with May-Grunwald stain and examined by light microscopy (magnification = 1250 ×). c & d, Cytoplasmic $IgG_{2b}$ paraprotein expression of cultured 5T33 murine myeloma cells. Cytoplasmic $IgG_{2b}$ expression was determined by indirect immunofluorescence as described in Materials and methods. Figure 1c demonstrates specific $IgG_{2b}$ cytoplasmic staining of the cultured 5T33 myeloma cells compared with background staining shown in Figure 1d (magnification = 300 ×).

### *Tumorigenicity of cultured 5T33 myeloma cells*

Cultured 5T33 myeloma cells were found to be highly tumorigenic in C57BL/KaLwRij mice with as few as 500 cells inducing paralysis and death as early as 36 days post-tumour inoculation (Figure 2). Survival time was directly related to tumour cell number. All of the animals injected with over $10^4$ cells developed paraprotein and paralysis resulting in death by day 50 post-tumour inoculation, whereas none of the animals injected with 100 cells showed any sign of disease by day 80 (Figure 2). No difference in tumour development was observed when tumour inoculation was performed via tail vein or jugular vein. The mean survival time of animals inoculated intrajugularly with $10^5$ 5T33 myeloma cells was 37.7 ± 2.3 days compared with 39.0 ± 3.1 days for animals inoculated via the tail vein ($P$ = NS). Paraprotein was detected 7–14 days prior to the onset of paralysis and increased with increasing tumour burden to a maximum of approximately 30 mg ml$^{-1}$ regardless of the initial cell dose (see below).

### *Kinetics and tissue distribution of cultured 5T33 myeloma cells*

Within two weeks of i.v. inoculation of $5 \times 10^5$ cultured 5T33 myeloma cells, a significant increase in the number of cytoplasmic $IgG_{2b}$ positive cells was observed in the liver and bone marrow of tumour-bearing animals compared with controls ($P < 0.01$, Figure 3, Table I). By day 21, all tissues examined demonstrated a large increase in cytoplasmic $IgG_{2b}$ positive cells when compared with control tissues ($P < 0.01$). The increased tumour cell population in the spleen and liver occurred in parallel with a significant increase in the size and weight of these two tissues ($P < 0.01$; Figure 4). The proportion of tumour cells in the various tissues continued to increase with time, particularly in the liver and bone marrow where, by week 5, over 70% of the isolated cells were positive for cytoplasmic $IgG_{2b}$. Similar liver, spleen and bone marrow involvement was observed when bone marrow cells from end-stage 5T33-bearing mice were used as the inoculum (data not shown).

Cytological evaluation of plasma cells in the various tissues demonstrated a similar pattern of tumour cell kinetics and distribution (Table I). Approximately 70% of the nucleated cells isolated from the blood of tumour-bearing animals at week 5 were positive for cytoplasmic $IgG_{2b}$ compared with 7.9 ± 4.3% in control animals ($n$ = 9, data not shown). $IgG_{2b}$

**Figure 2** Effect of 5T33 myeloma cell concentration on survival of C57BL/KaLwRij mice. Groups of 6–13 female mice were injected i.v. with 100 (▲), 500 (□), $10^3$ (■), $5 \times 10^3$ (◇), $10^4$ (◘), $10^5$ (◆) or $10^6$ (⊡) cultured 5T33 myeloma cells and monitored on a daily basis for the onset of paralysis. The data are presented as the percentage of animals surviving in each group with time (days) post-tumour inoculation.

**Table I** Tissue distribution of 5T33 myeloma cells following intrajugular inoculation

| | *Weeks post-tumour inoculation* 0 | 1 | 2 | 3 | 4 | 5 |
|---|---|---|---|---|---|---|
| | *Percentage cytoplasmic* $IgG_{2b}$ *positive cells* ( ± s.d.) | | | | | |
| I. Immunofluorescence | | | | | | |
| Thymus | <1 | <1 | 3.6 ± 0.5* | 5.5 ± 2.1* | 16.3 ± 2.3* | 26.2 ± 1.7* |
| Lymph-nodes | 19.1 ± 2.1 | 21.7 ± 3.7 | 21.8 ± 4.2 | 31.3 ± 4.7* | 37.1 ± 3.9* | 34.2 ± 5.0* |
| Spleen | 25.3 ± 2.3 | 28.5 ± 2.3 | 25.5 ± 2.7 | 51.2 ± 5.6* | 64.1 ± 4.7* | 57.9 ± 2.8* |
| Liver | 4.2 ± 1.8 | 10.6 ± 2.0 | 19.0 ± 2.0* | 54.0 ± 3.7* | 77.6 ± 3.0* | 74.9 ± 4.1* |
| Bone Marrow | 4.5 ± 1.8 | 7.6 ± 2.5 | 16.6 ± 2.7* | 64.2 ± 1.0* | 63.1 ± 3.4* | 71.1 ± 4.6* |
| II. Cytology | | | | | | |
| Thymus | <1 | 3.3 ± 0.8 | 2.0 ± 0.5 | 1.0 ± 0.0 | 12.8 ± 2.5 | 8.5 ± 2.5 |
| Lymph-nodes | <1 | 2.0 ± 0.4 | 2.0 ± 0.5 | 2.5 ± 0.8 | 15.3 ± 2.5* | 11.0 ± 2.1* |
| Spleen | <5 | 4.5 ± 0.6 | 5.7 ± 1.7 | 70.0 ± 4.9* | 71.8 ± 4.0* | 71.5 ± 4.1* |
| Liver | <1 | 1.0 ± 0.0 | 8.3 ± 0.8* | 46.0 ± 3.6* | 52.0 ± 2.9* | 42.5 ± 3.5* |
| Bone Marrow | <1 | 2.5 ± 0.8 | 13.3 ± 1.4* | 73.0 ± 3.4* | 78.8 ± 4.9* | 85.0 ± 5.7* |

Cytoplasmic $IgG_{2b}$ positive cells and plasma cells were identified as described in the Materials and methods. The data are presented as the mean ( ± s.d.) for 3–6 animals for each time point.
*$P < 0.05$ comparing the percentage of cytoplasmic $IgG_{2b}$ positive cells and plasma cells to baseline (week 0) values.

paraprotein was first detected in the blood between day 14 and day 21 post-tumour inoculation and increased significantly to a maximum of 30 mg $ml^{-1}$ by day 28 (Figure 5).

Alterations in normal blood cell profiles occurred in parallel with tumour progression. A significant elevation in platelet number was observed at day 7 post-tumour inoculation but by day 21, the cell populations of platelets, red blood cells and white blood cells were significantly reduced and remained low throughout the 5 week observation period ($P < 0.01$; Figure 6).

**Figure 3** Tissue distribution of 5T33 myeloma cells following intrajugular injection of 5 × $10^5$ cells. The data are presented as the mean ( ± s.d.) percent $IgG_{2b}$ cytoplasmic positive cells for 3–6 animals for each time point.

**Figure 4** Effect of 5T33 myeloma progression on liver and spleen weights. Mice were inoculated with 5T33 cultured myeloma cells as described in Figure 3. At weekly intervals, the liver and spleen were collected from at least three animals and weighed. The data is presented as the mean weight in grams ( ± s.d.) of the spleen and liver for 3–6 mice for each time point.

### Discussion

The 5T33 murine myeloma model is one of several multiple myelomas which arose spontaneously in aged C57BL/KaLwRij mice (Radl *et al.*, 1988). These tumours have been maintained by transplantation into syngeneic recipients and have been used for detailed studies on several aspects of the biology, histopathology and immunoregulation of myeloma (Radl *et al.*, 1985, 1988; Croese, 1987*a*; Croese *et al.*, 1987*b*; Croese *et al.*, 1991*a*, 1991*b*). These murine myelomas are remarkably similar to human multiple myeloma, and many of the findings may have direct clinical application.

Although this experimental animal model has proven invaluable for the *in vivo* study of myeloma, investigations have been limited by the lack of an *in vitro* cell line counterpart for this series of transplantable tumours. In particular, initial assessments of growth factor production and requirements for myeloma development cannot be evaluated *in vivo* or in short term *in vitro* cultures. Similarly, efficacy of the extensive range of chemical and biological agents now available for cancer therapy cannot be adequately assessed in an *in vivo* model due to the time and number of animals required to evaluate responses. We have established the 5T33 myeloma as an *in vitro* cultured cell line and characterised it *in vivo* to facilitate such studies.

**Figure 5** $IgG_{2b}$ paraprotein development with 5T33 tumour progression. Mice were inoculated with 5 × $10^5$ 5T33 cultured myeloma cells by intrajugular injection. At weekly intervals, blood was collected and analysed for $IgG_{2b}$ paraprotein by agarose gel electrophoresis (Materials and methods). The data are presented as the mean paraprotein concentration in milligrams $ml^{-1}$ ( ± s.d.) for 3–6 mice for each time point.

**Figure 6** Effect of 5T33 myeloma progression on blood cell profiles.

Early studies in our laboratory comparing the cultured 5T33 myeloma cells to those isolated from *in vivo* passaged tumours found the cells to be indistinguishable with respect to cytology and $IgG_{2b}$ paraprotein expression, and both parameters were consistent with the original description of the spontaneous tumour (Radl *et al.*, 1988). The morphological heterogeneity and variability in surface paraprotein expression which we observed in cultured 5T33 myeloma cells have also been described for the transplantable 5T2 multiple myeloma model where such heterogeneity was found to reflect different stages of differentiation (Croese, 1987*a*; Croese *et al.*, 1987*b*). This morphological heterogeneity of the 5T33 cultured cells may also explain the differences observed between the detection of cytoplasmic $IgG_{2b}$ positive cells by immunofluorescence and the cytological evaluation of plasma cells following 5T33 tumour inoculation (Table I). The smaller 5T33 myeloma cells were found to be intensely positive for the $IgG_{2b}$ paraprotein and yet did not appear cytologically as plasma cells. Conversely, the small percentage ($<10\%$) of 5T33 cells which were negative for cytoplasmic $IgG_{2b}$ were primarily of the larger 'plasma cell'-like population. Clonal analysis of the cultured 5T33 cells may help delineate the various precursor populations involved in myeloma development in terms of phenotype, proliferation potential, growth requirements and functional activities.

Tumorigenic potential of the cultured 5T33 myeloma cells is similar to the *in vivo* passaged tumour but, in our hands, paraprotein production, onset of paraplegia and survival time are much more consistent and reproducible when using cultured 5T33 cells than when using BM cells from tumour-bearing animals. This is probably due both to selection pressure of *in vitro* culture conditions on tumour subpopulations and to the variable number of tumour cells present in the BM of end-stage animals. We have shown in this study that the kinetics of 5T33 myeloma progression and tissue distribution *in vivo* are directly related to tumour cell dose. In addition, as few as 500 myeloma cells were found to be sufficient to induce malignancy which has important implications for clinical procedures utilising purged autologous bone marrow transplantation for the treatment of multiple myeloma. Based on this experimental evidence, purging procedures would have to achieve a bone marrow preparation containing less than 500 tumour cells to minimise recrudescence.

The extensive liver, spleen and bone marrow involvement observed during 5T33 tumour progression occurred whether using cultured 5T33 myeloma cells or *in vivo* passaged bone marrow cells as the inoculum. Spleen and bone marrow involvement has also been found for the 5T2 myeloma, but no liver involvement was described for this subline (Radl *et al.*, 1988). In our animals, liver involvement with 5T2 and 5T7 myeloma was not observed (unpublished data). The 5T33 myeloma did however involve the liver and may represent a different tumour type of myeloma. In the human disease, the type of myeloma has important implications in terms of prognosis and responsiveness to therapy (Croese, 1987*a*; Bartl *et al.*, 1982).

With the establishment of the 5T33 myeloma model as a cultured cell line, animals are no longer required for *in vivo* tumour maintenance thereby reducing animal use to direct experimental procedures. In addition, the development of this cultured cell line now allows a more detailed evaluation of myeloma cell susceptibility to a wide range of chemotherapeutic drugs and biological response modifiers. *In vitro* sensitivity studies using both human and animal tumour cell lines have been shown to be predictive of patient responsiveness, particularly in determining tumour resistance (van Hoff, 1990). The predictive value of *in vitro* sensitivity testing allows a systematic assessment of therapeutic options thereby providing a rational basis for treatment selection without placing patients at risk. Preliminary studies are underway using the cultured 5T33 myeloma cell line to evaluate the *in vitro* and *in vivo* susceptibility of this cell type to various forms of therapy including melphalan chemotherapy, internal radionuclide therapy using $^{153}$Samarium-ethylene-diamine-tetramethylene phosphonate, and immunotherapy using cytokines such as the interferons alpha, beta and gamma, tumour necrosis factor and the interleukins 1, 2 4, & 6.

The 5T33 cell line appears to be the first of this series of seven murine myelomas which does not require IL-6 for short- or long-term growth in culture (Dr J. Radl and Dr R. Mundy, personal communication). Other differences in growth factor production and/or requirements of myeloma cells are currently being evaluated using the cultured 5T33 cell line and early passages of 5T2 and 5T7 myeloma cells. Such differences may correlate with the degree of differentiation and pattern of growth in the bone marrow and therefore could be of direct clinical relevance. Collaborative studies have also been initiated to examine alterations in oncogene expression of the murine myeloma cell lines compared with that of their human counterparts.

Transplantable murine myeloma lines have allowed detailed study of the biology and histopathology of this type of malignancy. It is anticipated that our establishment of a well-characterised cultured cell line of 5T33 murine myeloma will provide specific information on growth factor requirements, therapeutic susceptibility and genetic alterations inherent in myeloma development with the potential for direct application to human multiple myeloma.

The authors thank Ms Maggie Wilson for her excellent technical assistance and Dr Robert Dunstan for his assistance with the FACS analyses. We also thank Dr Jiri Radl for providing the original *in vivo* passaged 5T33 myeloma in C57BL/KaLwRij mice. This work was supported in part by the Fremantle Hospital Research Foundation and the Cancer Foundation of Western Australia.

### References

ALEXANIAN, R. & DREICER, R. (1984). Chemotherapy of multiple myeloma. *Cancer*, **53**, 583–583.

BARLOGIE, B. (1991). Toward a cure for multiple myeloma? *New Engl. J. Med.*, **325**, 1304–1306.

BARLOGIE, B., EPSTEIN, J., SELVANAYAGAM, P. & ALEXANIAN, R. (1989). Plasma cell myeloma – new biological insights and advances in therapy. *Blood*, **73**, 865–879.

BARLOGIE, B., ALEXANIAN, R., DICKE, K.A., ZAGARS, G., SPITZER, G., JAGANNATH, S. & HORWITZ, L. (1987). High-dose chemoradiotherapy and autologous bone marrow transplantation for resistant multiple myeloma. *Blood*, **70**, 869–872.

BARTL, R., FRISCH, B. & BURKHARDT, R. (1982). Multiple myeloma. In *Bone Marrow Biopsies Revisited*; Bartl, R., Frisch, B. & Burkhardt, R. (eds). Basel: Karger. pp. 42–51.

CROESE, J.W., VISSINGA, C.S., BOERSMA, W.J.A. & RADL, J. (1991*a*). Immune regulation of mouse 5T2 multiple myeloma. I. Immune response to 5T2 MM idiotype. *Neoplasma*, **38**, 457–466.

CROESE, J.W. VAN DEN ENDEN-VIEVEEN, M.H.M. & RADL, J. (1991*b*). Immune regulation of 5T2 mouse multiple myeloma. II. Immunological treatment of 5T2 MM residual disease. *Neoplasma*, **38**, 467–474.

CROESE, J.W. (1987*a*). PhD Thesis: Experimental approaches to the treatment of multiple myeloma. Studies in an animal model. TNO Institute for Experimental Gerontology, Rijswijk, The Netherlands; Drukkerij J.H. Pasmans B.V.,'s – Gravenhage.

CROESE, J.W., VAS NUNES, C.M., RADL, J., VAN DEN ENDEN-VIEVEEN, M.H.M., BRONDIJK, R.J. & BOERSMA, W.J.A. (1987*b*). The 5T2 mouse multiple myeloma model: characterization of 5T2 cells within the bone marrow. *Br. J. Cancer*, **56**, 555–560.

HJORTH, M., HELLQUIST, L., HOLMBERG, E., MAGNUSSON, B., RODJER, S. & WESTIN, J. (1990). Initial treatment in multiple myeloma: no advantage of multidrug chemotherapy over melphalan-prednisone. *Br. J. Haematol.*, **74**, 185–195.

HUDSON, L. & HAY, F.C. (1980). *Practical Immunology*, 2nd edn. Oxford: Blackwell Scientific Publications.

JEPPSSON, J.O., LAURELL, C.B. & FRANZEN, B. (1979). Agarose gel electrophoresis. *Clin. Chem.*, **25**, 629–638.

KYLE, R., GREIPP, P.R. & GERTZ, M.A. (1986). Treatment of refractory multiple myeloma and considerations for future therapy. *Semin. Oncol.*, **13**, 326–333.

MCELWAIN, T.J. & ROWLES, R.L. (1983). High-dose intravenous melphalan for treatment of plasma-cell leukaemia and myeloma. *Lancet*, **ii**, 822–824.

MELLSTEDT, H., HOLM, G. & BJORKHOLM, M. (1984). Multiple myeloma, Waldenstrom's macroglobulinemia, and benign monoclonal gammopathy: characteristics of the B-cell clone, immunoregulatory cell populations, and clinical implications. *Adv. Cancer Res.*, **41**, 257–289.

RADL, J., CROESE, J.W., ZURCHER, C., VAN DEN ENDEN-VIEVEEN, M.H.M. & DE LEEUW, A.M. (1988). Multiple myeloma: animal model of human disease. *Am. J. Pathol.*, **132**, 593–597.

RADL, J., CROESE, J.W., ZURCHER, C., VAN DEN ENDEN-VIEVEEN, M.H.M., BRONDIJK, R.J., KAZIL, M., HAAIJMAN, J.J., REITSMA, P.H. & BIJVOET, O.L.M. (1985). Influence of treatment with APD-bisphosphonate on the bone lesions in the mouse 5T2 multiple myeloma. *Cancer*, **55**, 1030–1040.

VAN HOFF, D.D. (1990). He's not going to talk about *in vitro* predictive assays again, is he? *J. Natl. Cancer Inst.*, **82**, 96–101.